# Somatosensory abnormalities in Chinese patients with painful temporomandibular disorders

**DOI:** 10.1186/s10194-016-0632-y

**Published:** 2016-04-12

**Authors:** Guangju Yang, Lene Baad-Hansen, Kelun Wang, Kaiyuan Fu, Qiu-Fei Xie, Peter Svensson

**Affiliations:** Department of Prosthodontics and Center for Oral Functional Diagnosis, Treatment and Research, Peking University School and Hospital of Stomatology, Zhongguancun Nandajie 22, 100081 Beijing, China; Section of Orofacial Pain and Jaw Function, Department of Dentistry, Aarhus University, Scandinavian Center for Orofacial Neurosciences (SCON), Aarhus, Denmark; Center for Sensory-Motor Interaction (SMI), Department of Health Science and Technology, Aalborg University, Aalborg, Denmark; Department of Oral and Maxillofacial Radiology, Center for Temporomandibular Disorders and Orofacial Pain, Peking University School and Hospital of Stomatology, Beijing, China; Section of Orofacial Pain and Jaw Function, Department of Dentistry, Aarhus University, Aarhus, Denmark; Department of Dental Medicine, Karolinska Institutet, Scandinavian Center for Orofacial Neurosciences (SCON), Hudding, Sweden

**Keywords:** Temporomandibular disorders, Orofacial pain, Quantitative sensory testing, Somatosensory abnormality

## Abstract

**Background:**

The somatosensory phenotype of Chinese temporomandibular disorders (TMD) patients is not sufficiently studied with the use of contemporary techniques and guidelines.

**Methods:**

A standardized quantitative sensory testing (QST) battery consisting of 13 parameters with a stringent statistical protocol developed by the German Research Network on Neuropathic Pain was performed over the most painful and corresponding contralateral sites as well as the right hand of 40 Chinese patients with TMD and pain classified according to the Diagnostic Criteria for TMD (DC/TMD). The same QST protocol was performed bilaterally over the infraorbital, mental, and hand regions of 70 age- and gender-stratified healthy Chinese controls. Z-scores and loss/gain scores were computed for each TMD patient.

**Results:**

For patients, 82.5 % had somatosensory abnormalities in the painful facial region, while 60.0 % had abnormalities confined to the right hand. The most frequent abnormalities were somatosensory gain to pinprick (35.0 %) and pressure (35.0 %) stimuli, somatosensory loss to pinprick (25.0 %), cold (22.5 %), and heat (15.0 %) nociceptive stimuli. The most frequent loss/gain score was L0G2 (no somatosensory loss combined with a gain of mechanical somatosensory function) for both the facial (40.0 %) and hand (27.5 %) regions. Involving side-to-side differences in the evaluation increased the diagnostic sensitivity by 2.5–25.0 % across different parameters.

**Conclusions:**

Somatosensory abnormalities were commonly detected in Chinese TMD pain patients both within and outside the primary painful region, strongly indicating disturbances in the central processing of somatosensory stimuli. The individual variations in somatosensory abnormalities indicate a possible need for development of individualized TMD pain management.

**Electronic supplementary material:**

The online version of this article (doi:10.1186/s10194-016-0632-y) contains supplementary material, which is available to authorized users.

## Background

Temporomandibular disorders (TMD) pain is not only of major importance for individuals but also constitutes a major public health problem with a large impact on health-related expenses [1]. Despite the impact of TMD pain on the individual person and the community, studies indicate that patients with TMD pain are not sufficiently and adequately diagnosed or treated [2]. It is an open question whether the classification of pain syndromes based solely on the etiology is optimal, or whether it might be preferable to classify pain conditions on the basis of symptoms and signs [3, 4] or on patterns of somatosensory abnormalities [5, 6]. The individual pattern of somatosensory abnormalities at the affected and remote body areas reflects altered somatosensory functions. This may open a window to understand the mechanisms underlying pain.

Somatosensory sensitivity can be measured by quantitative sensory testing (QST) [7–12]. The German Research Network on Neuropathic Pain (DFNS) has established a standardized QST protocol for examination and data analysis [7, 13]. The DFNS introduced somatosensory profiles and the “loss/gain” coding system based on Z-scores computed using the means and standard deviations of reference data, and pain patients could be defined as different thermal and mechanical somatosensory abnormal groups according the coding system [7]. So far, just few studies have assessed somatosensory sensitivity in patients with TMD pain using the full standardized QST protocol. In one study, 21 patients with myofascial TMD pain were divided with respect to the tender point into an insensitive subgroup resembling healthy subjects and a sensitive subgroup resembling fibromyalgia syndrome patients’ QST profile [14]. The sensitive subgroup showed more expanded pain areas and generalized changes in pain perception over the cheek, trapezius, and hand dorsum in contrast to the insensitive patients with more localized changes [14]. Kothari et al. assessed somatosensory function at the temporomandibular joint (TMJ) and conditioned pain modulation of TMD pain patients with compared to healthy controls [15]. The results indicated that most (85.3 %) of the patients exhibited at least 1 or more somatosensory abnormalities at the painful TMJ with somatosensory gain with regard to pressure and punctate mechanical pain stimuli, and somatosensory loss with regard to mechanical detection and vibration detection stimuli [15]. The previous studies investigated the somatosensory changes of primarily Caucasian TMD pain patients, and showed that part of the patients had abnormal somatosensory function at the painful TMJ and in extra-trigeminal regions. However, the Chinese or eastern Asian populations are the biggest in the world but remain understudied using the contemporary and standardized QST protocols [7].

The aim of this study was to expand the description of the clinical phenotype and to evaluate somatosensory abnormalities in painful facial and remote regions of Chinese patients with TMD pain diagnosed with reproducible and validated criteria according to the international Diagnostic Criteria for TMD [16]. The TMD pain patients were compared with an age-, gender- and region-stratified healthy group according to the DFNS method.

## Methods

### Participants

#### Healthy participants

Healthy Chinese participants between 18 and 70 years of age were recruited through advertisement in the community. Exclusion criteria were: ongoing pain or reported chronic pain in the last 6 months; serious systemic disease or previous radiotherapy or chemotherapy; intake of medicine affecting the central nervous system; fibromyalgia syndrome, or self-reported psychogenic illness. Individuals’ medical history was checked by a clinical dentist and one TMD specialist performed DC/TMD on all the participants to exclude TMD patients. One hundred and three healthy Chinese volunteers responded to the advertisement. Finally, 70 healthy participants between 24 and 69 years of age (42.3 ± 12.5 years), 36 females (43.1 ± 12.8 years) and 34 males (41.5 ± 12.3 years) met the criteria, were recruited, and finished the whole test.

#### TMD pain patients

From 2012 to 2014, Chinese individuals with a primary complaint of pain in the orofacial region were recruited from the Center for TMD and Orofacial Pain of Peking University School and Hospital of Stomatology, China. All patients were investigated and diagnosed (myalgia or arthralgia) by one TMD specialist who had received extensive training and calibration in the use of the Diagnostic Criteria for Temporomandibular Disorders (DC/TMD) [16]. Pain intensity just before the test was rated by the patients on a 0–10 cm visual analogue scale (0 = “no pain”, 10 = “most pain imaginable”). Exclusion criteria were: serious systemic disease or previous radiotherapy or chemotherapy; intake of medicine affecting the central nervous system; fibromyalgia syndrome, headache, or any therapy during the 2 weeks prior to inclusion, bilateral TMD pain. Of the 960 Chinese patients, most were excluded either because they suffered from additional painful conditions or multiple disorders affecting the nervous system, they were not interested in the study, or their records were incomplete. Forty patients with TMD pain (8 males, 32 females) aged 20–77 years (44.3 ± 15.5 years, ≤40 year *n* = 19, >40 year *n* = 21) were finally recruited and finished the test. The patients were diagnosed using DC/TMD as having myalgia (*n* = 30, temporalis and masseter muscle origin) and arthralgia without intra-articular joint disorders (*n* = 10). The self-reported TMD peak pain intensity before the test on the 0–10 cm scale was 2.9 ± 1.7 cm. The range of self-reported pain duration was 14.5 ± 21.1 months (0.25–100 months, <3 months *n* = 11, ≥3 months *n* = 29). The psychological status of patients was evaluated using the SCL-90 scale with 9 domains [17].

The study adhered to the Declaration of Helsinki II, was approved by the local Ethics Committee (PKUSSIRB-2013012), and all participants gave written informed consent.

### Quantitative Sensory Testing (QST) protocol

**Fig. 1:**
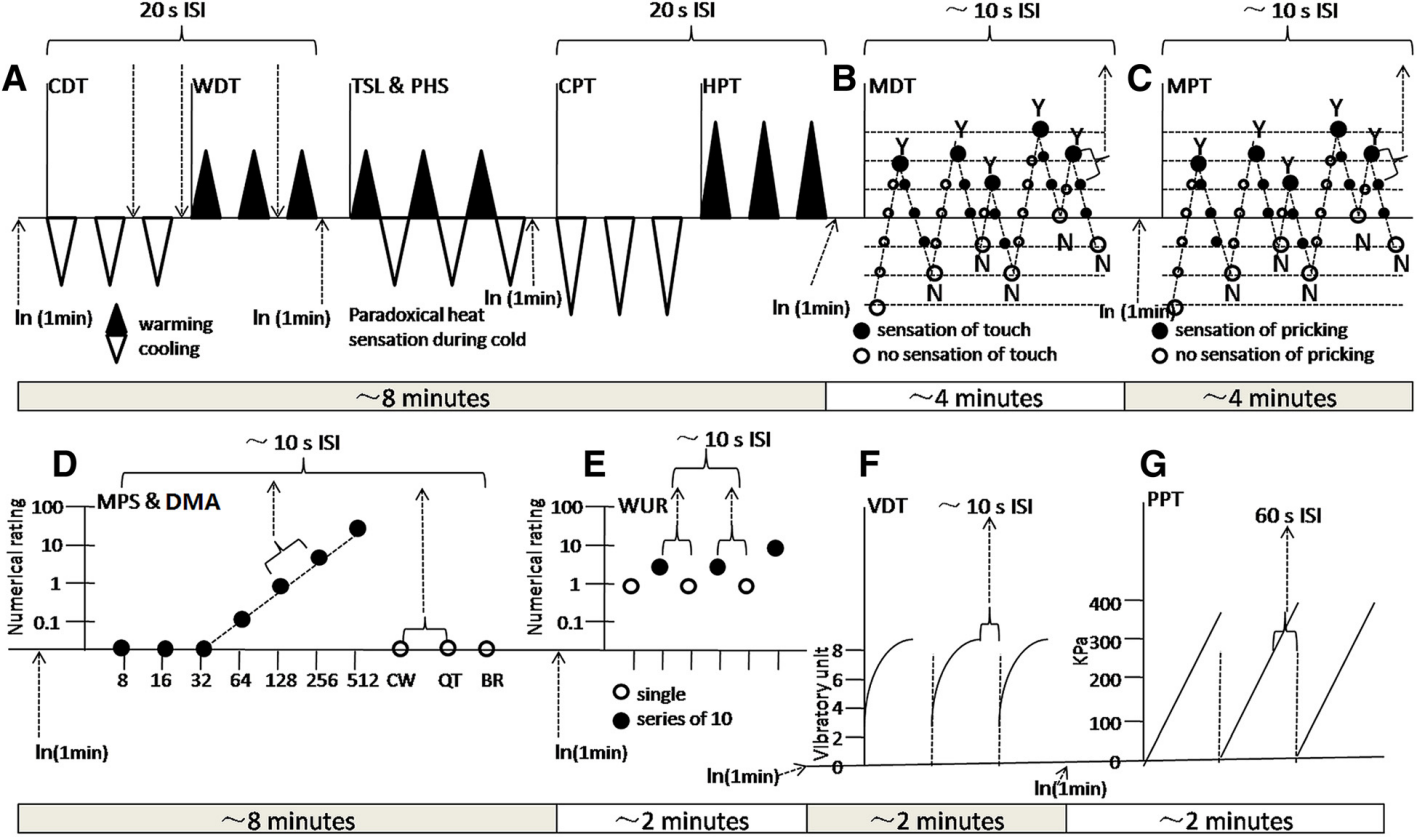
The battery of quantitative sensory testing (QST). The standardized QST protocol consists of 7 tests (A-G) to assess the 13 parameters. **a** Thermal testing comprises detection and pain thresholds for cold, warm and hot stimuli (C and A-delta fiber mediated): cold detection threshold (CDT), warm detection threshold (WDT), cold pain threshold (CPT) and heat pain threshold (HPT) with inter-stimulus interval of 20 s; number of paradoxical heat sensations (PHS) during the thermal sensory limen procedure (TSL) for alternating warm and cold stimuli. **b** Mechanical detection threshold (MDT) test using von Frey-filaments (A-beta fiber mediated) with inter-stimulus interval of ~10 s. **c** Mechanical pain threshold (MPT) for pinprick stimuli (mediated by A-delta fiber) assessing hyper- or hypoalgesia with inter-stimulus interval of ~10 s. **d** Stimulus–response-functions: mechanical pain sensitivity (MPS) assess A-delta fiber mediated sensitivity to sharp stimuli (pinprick) and dynamic mechanical allodynia (DMA) assess A-beta fiber mediated pain sensitivity to stroking light touch (CW = cotton wisp; QT = cotton wool tip; BR = brush), with inter-stimulus interval of ~10 s. **e** Wind-up ratio (WUR) compares the numerical ratings within three trains of a single pinprick stimulus (**a**) with a series (**b**) of 10 repetitive pinprick stimuli to calculate WUR as the ratio: *b*/*a,* with ~10 s intervals between single and series stimulus. **f** Vibration detection threshold (VDT) tests for A-beta fiber function using a Rydel–Seiffer 64 Hz tuning fork with intervals of ~10 s. **g** Pressure pain threshold (PPT) is the only test for deep pain sensitivity, most probably mediated by muscle C- and A-delta fibers, with inter-stimulus interval of 60 s. ISI = Inter-Stimulus-Interval, In = Instruction

The standardized QST battery developed by DFNS and modified for the trigeminal region was used in this study [8–12, 15]. All QST measures were performed in a quiet room at 21–23 °C. The QST protocol consists of 7 tests measuring a total of 13 thermal and mechanical parameters: A. Thermal testing comprises detection and pain thresholds for cold, warm, and hot stimuli (mediated by C- and A-delta fibers): cold detection threshold (CDT); warm detection threshold (WDT); number of paradoxical heat sensations (PHS) during the thermal sensory limen procedure (TSL) for alternating warm and cold stimuli; cold pain threshold (CPT); and heat pain threshold (HPT). B. Mechanical detection threshold (MDT) tests for A-beta fiber function using von Frey filaments. C. Mechanical pain threshold (MPT) tests for A-delta fiber-mediated hyper- or hypo-algesia to pinprick stimuli. D. Stimulus–response-functions: mechanical pain sensitivity (MPS) to pinprick stimuli and dynamic mechanical allodynia (DMA) assessment of A-delta fiber-mediated sensitivity to sharp stimuli (pinprick), as well as A-beta fiber-mediated pain sensitivity to stroking light touch (CW, cotton wisp; QT, cotton-wool tip; BR, brush). E. Wind-up ratio (WUR) compares the numerical ratings within three trials of a single pinprick stimulus (a) with a series (b) of 10 repetitive pinprick stimuli to calculate WUR as the ratio b/a. F. Vibration detection threshold (VDT) tests for A-beta fiber function using a Rydel–Seiffer 64-Hz tuning fork. G. Pressure pain threshold (PPT) is the only test for deep-pain sensitivity, most probably mediated by muscle C- and A-delta fibers [13, 15]. The investigator in this study was carefully instructed and trained according to the latest guidelines [9]. The full QST protocol took approximately 30 min per test site (Fig. 1). All the tests were performed following the sequence suggested by DFNS.

In the present study, three skin regions of TMD pain patients were examined: the painful facial region, the mirror region on the contralateral side, and the dorsum of the right hand. The contralateral sites were tested first, followed by the painful sites. Healthy participants were investigated bilaterally on six skin regions: the infraorbital regions (V2), the mental regions (V3), and the dorsum of the hands. A standard set of instructions lasting ~1 min was read to the participants for each different modality just before the beginning of each test, i.e. there were 1-min intervals between tests [10–13].

#### Thermal thresholds and thermal sensory limen

Thermal testing was performed using Medoc Pathway (Medoc Ltd, Israel) with an Advanced Thermal Stimulator (30 mm × 30 mm) [10–13]. CDT, WDT, CPT, and HPT were measured in triplicate [10–13]. For the TSL, the temperature first went up, and the participants pressed a button when they perceived a change [10–13]. The number of PHSs during this procedure was recorded [10–13]. Baseline temperature was set at 32 °C for all thermal testing, ramped stimuli of 1 °C/s was used, cutoff temperatures were set at 0 and 50 °C [10–13].

#### Mechanical detection threshold

The MDT was measured with a standard set of Semmes-Weinstein monofilaments (Touch Test TM Sensory Evaluator, North Coast Medical, Inc., Morgan Hill, CA) with 20 different diameters [10–12, 15]. Five repeated threshold measurements were made, each by applying a series of ascending and descending stimulus intensities; the final threshold was the geometric mean of the five series [10–13].

#### Mechanical pain threshold, mechanical pain sensitivity to pinprick stimuli, dynamic mechanical allodynia, and wind-up ratio for repetitive pinprick stimuli

Weighted pinprick stimuli were delivered with seven custom-made punctate mechanical stimulators with fixed stimulus intensities (flat contact area 0.2 mm in diameter) that exerted forces of 8 to 512mN to determine the MPT [10–13]. The method of limits, which was used to determine the MDT, was also used to determine the MPT [10–13].

MPS and DMA were evaluated using two sets of instruments in a stimulus–response assessment [7, 13]. To determine MPS, 7 weighted pinprick stimulators were used (as for MPT). Three tactile stimulators were used to determine DMA: a cotton wisp (~3mN), a cotton-wool tip (Q-tip, ~100mN) attached to a flexible handle, and a disposable toothbrush (Top Dent®, Meda AB, Solna, Sweden, ~200-400mN) [10–12, 15]. A series of 10 measurements was made three times, each with the 10 stimulators (7 pinpricks and 3 tactile stimulators) applied in a different order, as specified in the DFNS protocol [10–13]. For each of the resulting 30 stimuli, the participant chose a pain rating on a 0 to 100 scale with the endpoints ‘0’ indicating “no pain” and ‘100’ indicating “most intense pain imaginable”.

To measure the WUR for repetitive pinprick stimuli, the perceived magnitude of a train of 10 pinprick stimuli repeated at 1 Hz was divided by that of a single pinprick stimulus with the same force [10, 13]. The WUR test was repeated three times [10–13, 15].

#### Vibration detection threshold

The vibration detection threshold (VDT) was measured tuning fork (64 Hz, 8/8 scale) [7, 13]. VDT was performed on bony prominences bilaterally for each participant: the zygomatic process, the lower edge of the mandible, and the ulnar styloid process. The participant indicated when the vibration could no longer be sensed on a 9-point (0–8) scale [10–13, 15]. The test was repeated three times.

#### Pressure pain threshold

The pressure pain threshold (PPT) was measured using a computerized pressure algometer (Medoc AlgoMed, Israel) [10–13, 15]. PPT was measured on the painful site, the corresponding contralateral site, and the right thenar muscle of patients; and on the temporalis, masseter, and thenar muscles bilaterally of healthy participants; both with a constant application rate of 30kPa/s [10–13, 15]. The test was repeated three times.

### Data analysis and statistics

All absolute QST scores are presented as mean ± SD. Cold and heat pain thresholds as well as vibration thresholds were normally distributed. All other parameters were normally distributed only after log-transformation. There was no PHS or DMA in the healthy group. For the remaining 11 parameters, the data for healthy participants were considered as reference values. As our previous study showed, somatosensory data from the 70 healthy participants exhibited significant gender, age, and region differences [11, 15]. The data were stratified for age group (younger ≤40 years, *n* = 32; older >40 years, *n* = 38), gender, and region (infraorbital, mental, and hand) to allow a more detailed analysis of the somatosensory abnormalities of TMD pain patients (absolute data in Additional file 1: Table S1, side-to-side differences data in Additional file 1: Table S2).

#### Z-transformation of QST data

A z-transformation was performed for each variable. The sign of the resulting z-score was adjusted in such a way that those above >0 indicated a gain-of-function when the participant was more sensitive to the stimuli compared with controls (hyperesthesia, hyperalgesia, and allodynia), while z-scores <0 indicated a loss-of-function referring to a lower sensitivity (hypoesthesia and hypoalgesia) [7, 18]. A z-score of 0 ± 1.96 represents the range that can be expected to include 95 % of the control data [7, 18]. To compare individual QST data from patients with the mean reference range of the same region (V2, V3, or hand) of age- and gender-matched controls, the patient data were z-transformed for each single variable in the same way, using the transformation parameters of the reference group. The individual pain site z-scores were calculated as (mean_reference group_ ‐ individual value)/SD_reference group_ [4, 10]. Z-scores >1.96 and < −1.96 indicate values outside of the 95 % CI of the reference group data. Such values were considered to be “absolute abnormalities” [7]. Also, the side-to-side differences of each QST parameter from patients were compared with the 95 % CI of the side-to-side differences of the reference group [7]. If the side-to-side differences were larger than the upper limit of the 95 % CI of the reference group, the value was considered to be a “relative abnormality” [7]. In accordance with Maier et al. [7], the assessment of frequencies of loss and gain of somatosensory function included a combination of absolute and relative (side-to-side) abnormalities (the basis of the loss/gain coding system).

#### Assessment of somatosensory loss and gain of function

The loss/gain coding system was applied [7, 18]. The loss/gain score combines a score of somatosensory loss of function (L0, L1, L2, or L3) with a score of somatosensory gain of function (G0, G1, G2, or G3) [7, 18]. The number after the letter L or G indicates whether the abnormality is related to the thermal modalities alone (1), the mechanical modalities alone (2), or mixed (3) (thermal and mechanical). If measures of thermal and/or mechanical detection (CDT, WDT, TSL, MDT, or VDT) were abnormal on the affected side in comparison with the reference data (“absolute abnormality”) or if abnormally large side-to-side differences were detected (“relative abnormality”), it was recorded as one of the following: L1, isolated loss of small fiber function (if abnormal thermal detection thresholds [CDT, TSL, or WDT] alone); L2, isolated loss of large fiber function (if abnormal mechanical detection thresholds [MDT or VDT] alone); or L3, mixed loss of function (if loss of both small and large fiber function) [7, 18]. Likewise for somatosensory gain, thermal hyperalgesia (G1) was recorded if gain-of-function in cold or heat pain thresholds (CPT or HPT) were found (absolute or relative abnormality). Mechanical hyperalgesia (G2) was recorded if gain-of-function (absolute or relative abnormality) was detected for MPT, MPS, or PPT, or if the DMA score exceeded 0. Mixed gain (G3) was recorded in individuals with gain of both thermal and mechanical somatosensory function. L0 was scored if no loss of somatosensory function was presented, and G0 if no gain of somatosensory function was detected.

#### Statistics

All statistical calculations were performed using SPSS 17.0 for Windows (IBM, Armonk, New York City, NY). The distribution of frequencies of loss and gain of somatosensory function at the painful site and hand between groups according to loss/gain coding was evaluated with *χ*^2^ tests with Bonferroni adjustments for multiple comparisons. Values of *P* <0.01 were considered statistically significant.

## Results

### Somatosensory abnormalities in healthy participants

As expected due to natural variation, a few abnormalities were found in the reference group (mean across parameters for somatosensory loss 4.0 ± 2.0 % and for somatosensory gain 1.1 ± 2.2 %) (Table 1) [7, 18].

**Table 1:** Mean and standard deviation (SD) of the quantitative sensory testing (QST) parameters from the infraorbital, mental, and hand regions before and after z-transformation in the reference group and from the painful site in the temporomandibular disorders (TMD) patients

		Reference group (420 sites^a^)					TMD patient group (40 sites)			
	Absolute mean (SD) (140 sites/region^a^)			z-scores mean (SD)	<−1.96	>1.96	Absolute mean (SD)	z-scores mean (SD)	<−1.96	>1.96
	Infraorbital	Mental	Hand		*n* (%)	*n* (%)			*n* (%)	*n* (%)
CDT	−0.90(0.46)	−0.93(0.38)	−1.72(1.04)	0.00(1.00)	22(5.2 %)	0(0.0 %)	−0.84(0.34)	0.10(1.06)	2(5.0 %)	0(0.0 %)
WDT	1.16(0.35)	1.34(0.57)	2.50(1.25)	0.00(1.00)	21(5.0 %)	0(0.0 %)	1.14(0.46)	0.31(1.07)	1(2.5 %)	1(2.5 %)
TSL	2.80(1.38)	2.68(1.30)	4.95(2.55)	0.00(1.00)	21(5.0 %)	0(0.0 %)	2.41(1.20)	0.02(1.11)	2(5.0 %)	0(0.0 %)
CPT	23.82(5.86)	23.51(6.81)	23.43(6.92)	0.00(1.00)	20(4.7 %)	0(0.0 %)	22.20(7.68)	−0.29(1.14)	6(15.0 %)	0(0.0 %)
HPT	37.61(2.75)	38.99(3.19)	40.52(3.55)	0.00(1.00)	16(3.8 %)	0(0.0 %)	38.71(3.32)	−0.07(1.10)	4(10.0 %)	0(0.0 %)
MDT	0.13(0.08)	0.12(0.09)	3.05(3.37)	0.00(1.00)	23(5.5 %)	0(0.0 %)	0.15(0.25)	−1.42(8.12)	4(10.0 %)	0(0.0 %)
MPT	89.19(66.94)	78.57(73.68)	155.33(110.58)	0.00(1.00)	22(5.2 %)	0(0.0 %)	78.46(86.08)	−0.14(1.58)	4(10.0 %)	0(0.0 %)
MPS	2.08(1.53)	2.23(1.72)	1.27(1.30)	0.00(1.00)	0(0.0 %)	24(5.7 %)	4.95(4.64)	1.69(3.21)	0(0.0 %)	14(35.0 %)
WUR	2.99(1.90)	3.02(2.10)	2.90(1.88)	0.00(1.00)	0(0.0 %)	25(5.9 %)	3.40(2.01)	0.26(1.06)	0(0.0 %)	3(7.5 %)
VDT	7.36(0.48)	7.49(0.55)	7.65(0.43)	0.00(1.00)	19(4.5 %)	0(0.0 %)	7.63(0.41)	0.22(1.02)	2(5.0 %)	0(0.0 %)
PPT	170.94(56.84)	144.74(48.75)	239.60(103.21)	0.00(1.00)	22(5.2 %)	0(0.0 %)	68.82(26.43)	1.59(0.60)	0(0.0 %)	11(27.5 %)
ALL					Mean 16.9(4.0 %)	Mean 4.45(1.1 %)			Mean 2.27(5.7 %)	Mean 2.63(6.6 %)

The individual z-scores for each parameter were calculated as (mean_reference group_ ‐ individual value)/SD_reference group_ with regard to data stratified according to gender, age, and region [10, 13]. Z-scores above 1.96 and below −1.96 indicate values outside of the 95 % confidence interval (CI) of the reference group data. Such values were considered to be absolute abnormalities

*CDT* cold detection threshold, *WDT* warmth detection threshold, *TSL* thermal sensory limen, *PHS* paradoxical heat sensation, *CPT* cold pain threshold, *HPT* heat pain threshold, *MDT* mechanical detection threshold, *MPT* mechanical pain threshold, *MPS* mechanical pain sensitivity, *DMA* dynamic mechanical allodynia, *WUR* windup ratio, *VDT* vibration detection threshold, *PPT* pressure pain threshold

^a^ The infraorbital, mental and hand regions were measured bilaterally in each healthy participant, 420 sites were tested for 70 participants, and 140 test sites for each region

### Absolute abnormalities of QST z-scores and side-to-side differences in TMD pain patients

**Fig. 2:**
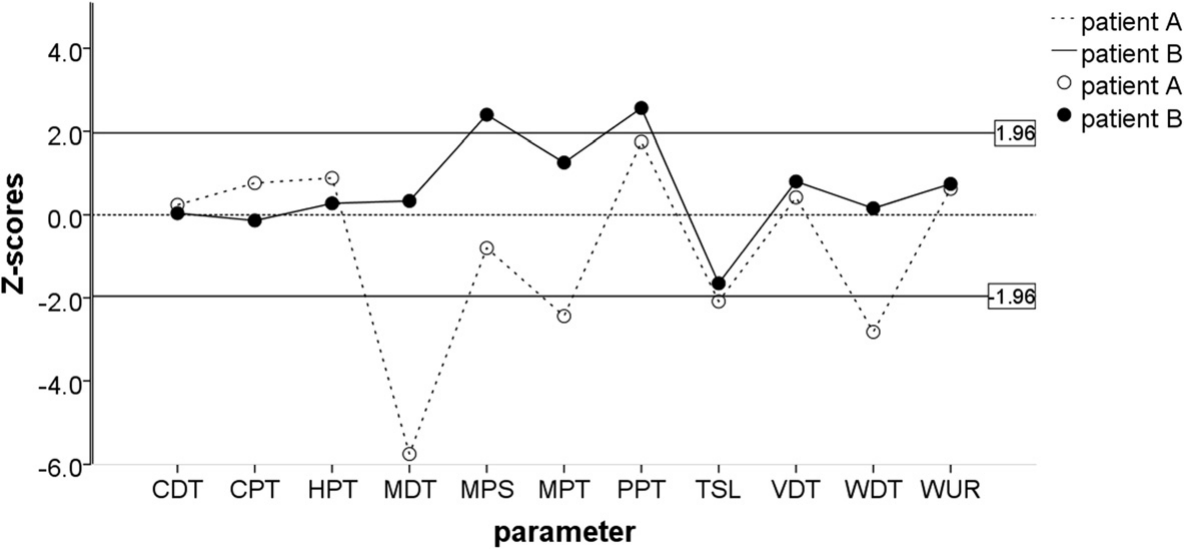
Examples of somatosensory z-score profiles of 2 patients with painful temporomandibular disorders indicating abnormalities involving different peripheral or central pain mechanisms [10–13]. Open symbols indicate patient A (loss of function to tactile, pinprick, and thermal non-nociceptive stimuli), and closed symbols indicate patient B (gain of function to painful pinprick and pressure stimuli). The zone between the two lines (−1.96 < z < 1.96) is the normal range based on the healthy material. CDT: cold detection threshold; WDT: warmth detection threshold; TSL: thermal sensory limen; CPT: cold pain threshold; HPT: heat pain threshold; MDT: mechanical detection threshold; MPT: mechanical pain threshold; MPS: mechanical pain sensitivity; WUR: windup ratio; VDT: vibration detection threshold; PPT: pressure pain threshold

There was no PHS or DMA in the patient group. The most frequent somatosensory absolute abnormalities at the painful site of the TMD pain group was (in order of frequency): somatosensory gain with regard to MPS, PPT, WUR, and WDT; and somatosensory loss with regard to CPT, HPT, MDT, MPT, CDT, TSL, VDT, and WDT (Table 1, Fig. 2).

**Fig. 3:**
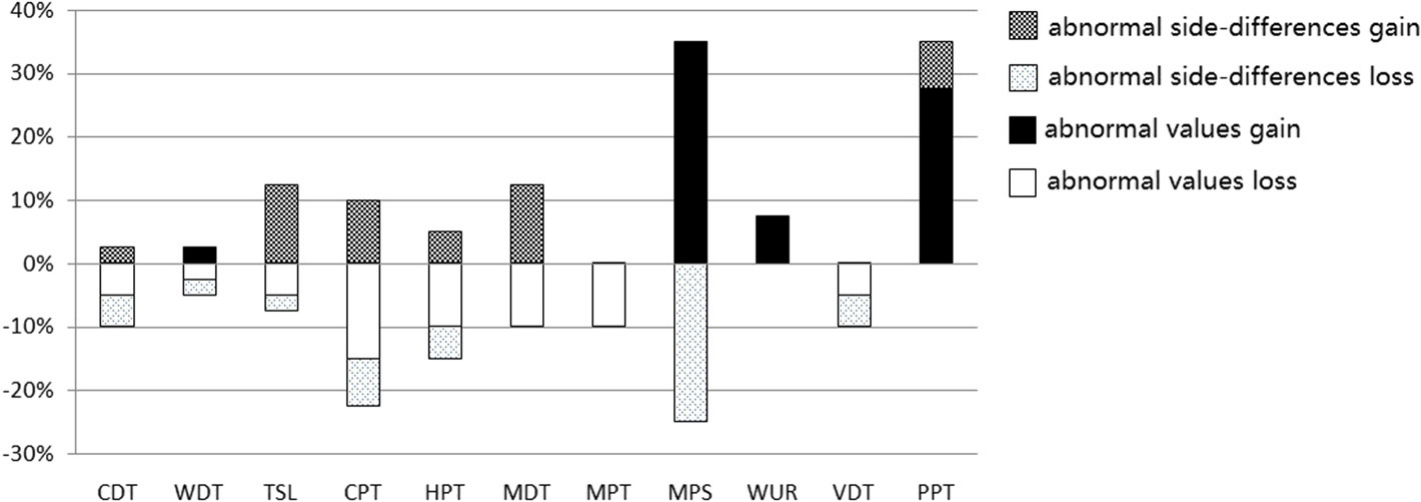
Absolute and relative abnormalities for temporomandibular disorder patients in the painful area. Values outside the 95 % confidence intervals of the healthy reference data are considered to be absolute abnormalities, and differences of the affected side versus the unaffected side outside the 95 % confidence intervals of such differences of the healthy reference data are considered to be relative abnormalities. The y-axis shows the percentage of patients (*n* = 40), with positive sensory signs plotted upwards and negative sensory signs plotted downwards. CDT: cold detection threshold; WDT: warmth detection threshold; TSL: thermal sensory limen; CPT: cold pain threshold; HPT: heat pain threshold; MDT: mechanical detection threshold; MPT: mechanical pain threshold; MPS: mechanical pain sensitivity; WUR: windup ratio; VDT: vibration detection threshold; PPT: pressure pain threshold

The frequencies of abnormal values in the painful region for each QST parameter in 40 TMD pain patients are shown in Fig. 3. For all non-nociceptive detection thresholds except for WDT, only sensory loss was detected according to the absolute data (roughly 5–10 % across different parameters) (Table 1). Side-to-side differences identified additional patients with relative sensory loss to non-nociceptive stimuli (for different parameters between 2.5 and 5.0 % additional patients), and relative sensory gain were detected for the non-nociceptive parameters (for different parameters between 2.5 and 12.5 % additional patients) (Fig. 3 and Table 2). For the nociceptive parameters, both sensory loss (hypoalgesia) (10.0–15.0 %) and sensory gain (hyperalgesia) (7.5–35.0 %) were found in the absolute data (Table 1 and Fig. 3). The inclusion of abnormal side-to-side differences increased both the frequency of patients with loss (5.0–25.0 % additional patients with hypoalgesia) and with gain (5.0–10.0 % additional patients with hyperalgesia) in nociceptive function (Fig. 3 and Table 2).

**Table 2:** Mean values and standard deviation (SD) of absolute values of side-to-side differences at the painful sites in the temporomandibular disorders (TMD) patients and the healthy reference group

	Reference group						TMD patients group	
	Infraorbital mean(SD)	95%CI (upper limit)	Mental mean(SD)	95%CI (upper limit)	Hand mean (SD)	95%CI (upper limit)	Painful site mean(SD)	>95%CI
								*n* (%)
CDT	0.23(0.20)	0.27	0.20(0.22)	0.26	0.44(0.53)	0.57	0.19(0.16)	3(7.5 %)
WDT	0.23(0.22)	0.28	0.32(0.31)	0.39	0.86(0.80)	1.05	0.19(0.19)	1(2.5 %)
TSL	0.64(0.49)	0.76	0.43(0.38)	0.52	1.32(1.52)	1.68	0.59(0.67)	6(15.0 %)
CPT	1.86(1.72)	2.27	1.33(1.50)	1.69	2.11(2.11)	2.62	2.84(3.97)	7(17.5 %)
HPT	1.43(1.63)	1.82	1.16(1.07)	1.42	1.12(0.93)	1.35	1.14(1.08)	4(10.0 %)
MDT	0.05(0.07)	0.06	0.04(0.08)	0.06	2.35(2.88)	3.04	0.07(0.19)	5(12.5 %)
MPT	33.21(37.38)	42.12	27.52(50.91)	39.66	51.64(49.74)	63.50	23.74(40.88)	0(0.0 %)
MPS	0.60(0.90)	0.82	0.73(0.95)	0.95	0.50(0.74)	0.68	1.90(2.38)	10(25.0 %)
WUR	1.00(1.27)	1.30	1.02(1.28)	1.32	0.91(1.02)	1.16	0.70(0.86)	0(0.0 %)
VDT	0.24(0.22)	0.29	0.16(0.18)	0.21	0.18(0.18)	0.22	0.24(0.24)	2(5.0 %)
PPT	24.64(23.85)	30.33	25.71(21.62)	30.87	49.09(41.50)	58.99	38.19(38.41)	3(7.5 %)

*CDT* cold detection threshold, *WDT* warmth detection threshold, *TSL* thermal sensory limen, *PHS* paradoxical heat sensation, *CPT* cold pain threshold, *HPT* heat pain threshold, *MDT* mechanical detection threshold, *MPT* mechanical pain threshold, *MPS* mechanical pain sensitivity, *DMA* dynamic mechanical allodynia, *WUR* windup ratio, *VDT* vibration detection threshold, *PPT* pressure pain threshold, *CI* confidence interval

The upper limit of the 95 % CI (95 % CI_up_) of the side-to-side differences in the reference group is also given as it was used in the evaluation of relative abnormalities for the LossGain scores. The abnormal frequencies of TMD patients were evaluated by age, gender and site stratified data

### Somatosensory abnormalities of TMD pain patients according to the loss/gain coding system

The distribution of participants in each group according to the loss/gain coding system is shown in Table 3. Only 17.5 % of the pain patients had no somatosensory abnormalities, compared with 68.8 % of the reference group (*P* <0.001). L0G2 (no somatosensory loss with gain of mechanical somatosensory function) was the most frequent coding in the TMD group (40.0 %), which was significantly different from the reference group (10.2 %) (*P* <0.001). The cumulative proportion of somatosensory loss without any gain (L1G0, L2G0, and L3G0) was 12.5 % in the TMD group and 19.7 % in the reference group (*P* = 0.399). The cumulative proportion of participants presenting with somatosensory gain without any loss (L0G1, L0G2, and L0G3) was higher in the TMD group (50.0 %) than in the reference group (10.2 %) (*P* <0.001). The cumulative proportion of the groups showing mixed loss and gain (L1G1, L1G2, L1G3, L2G1, L2G2, L2G3, L3G1, L3G2, or L3G3) was higher in the TMD group (20.0 %) than in the reference group (1.2 %) (*P* <0.001) (Table 3).

**Table 3:** Loss and gain distribution in the painful region in temporomandibular disorder (TMD) patients and the healthy reference group

Loss	Gain				All
	G0 (None)	G1 (Thermal)	G2 (Mechanical)	G3 (Both)	
TMD patients (40 sites)					
L0 (None)	7(17.5 %)	1(2.5 %)	16(40.0 %)	3(7.5 %)	27(67.5 %)
L1 (Thermal)	2(5.0 %)	0(0.0 %)	3(7.5 %)	0(0.0 %)	5(12.5 %)
L2 (Mechanical)	2(5.0 %)	0(0.0 %)	3(7.5 %)	0(0.0 %)	5(12.5 %)
L3 (Both)	1(2.5 %)	0(0.0 %)	1(2.5 %)	1(2.5 %)	3(7.5 %)
All	12(30.0 %)	1(2.5 %)	23(57.5 %)	4(10.0 %)	40(100 %)
Reference group (420 sites)					
L0 (None)	289(68.8 %)	0(0.0 %)	43(10.2 %)	0(0.0 %)	332(79.0 %)
L1 (Thermal)	42(10.0 %)	0(0.0 %)	4(1.0 %)	0(0.0 %)	46(11.0 %)
L2 (Mechanical)	38(9.0 %)	0(0.0 %)	1(0.2 %)	0(0.0 %)	39(9.3 %)
L3 (Both)	3(0.7 %)	0(0.0 %)	0(0.0 %)	0(0.0 %)	3(0.7 %)
All	372(88.6 %)	0(0.0 %)	48(11.4 %)	0(0.0 %)	70(100 %)

Sensory abnormality coding system [7]: hypoesthesia to thermal stimuli (loss of detection in the cold or warm detection threshold) was coded as L1, and hypoesthesia to mechanical stimuli (loss of detection in mechanical or vibration detection threshold) as L2. Signs of hyperalgesia to thermal stimuli (gain-of-function in heat or cold pain threshold) were coded as G1, and hyperalgesia to mechanical stimuli (gain-of-function in mechanical pain threshold or sensitivity, dynamic mechanical allodynia, or pressure pain threshold) as G2. When both thermal and mechanical abnormalities were present, L3 or G3 were defined. Normal values were coded as zero

### Somatosensory abnormalities in the hand region

The individual hand dorsum z-scores for each modality based on the means and SDs of the healthy reference data showed that 25.0 % of patients had gain of somatosensory function for MPS, 20.0 % had gain of function for WUR; 12.5 % had somatosensory loss for MPT, 10.0 % had loss of function for CDT, WDT, and PPT, and 7.5 % had loss of function for TSL and CPT (Additional file 1: Table S3).

The distribution of the participants in each group according to the loss/gain coding system is shown in Additional file 1: Table S4. Forty percent of the TMD pain patients had no somatosensory abnormalities in the hand region, compared with 75.7 % of the reference group (*P* <0.001). L0G2 was the most frequent coding in the TMD pain group (27.5 %), which was markedly higher than in the healthy group (4.3 %) (*P* = 0.001). The cumulative proportion of somatosensory loss without any gain (L1G0, L2G0, and L3G0) was 15.0 % in the TMD group and 17.1 % in the reference group (*P* = 0.771). The cumulative proportion of participants presenting with somatosensory gain at the hand site without any loss (L0G1, L0G2, and L0G3) was higher in the TMD group (30.0 %) than in the reference group (4.3 %) (*P* <0.001). The cumulative proportion in the groups showing mixed loss and gain (L1G1, L1G2, L1G3, L2G1, L2G2, L2G3, L3G1, L3G2, or L3G3) in the hand region was higher in the TMD group (15.0 %) than in the reference group (2.9 %) (*P* = 0.026) (Additional file 1: Table S4).

### Psychological status of patients

Twelve of 40 patients (30 %) had psychological abnormalities compared with reference data [17]. Somatization was the most frequent psychological changes (25 %), Paranoid ideation was the lowest (10 %) (Additional file 1: Table 4).

**Table 4:** Psychological status of Chinese TMD pain patients

Categories	n^a^/(%)	Patient (mean ± SD)	Control (mean ± SD)
Somatization	10 (25 %)	1.97 ± 0.67	1.37 ± 0.48
Obsessive compulsive	7 (17.5 %)	2.00 ± 0.84	1.62 ± 0.58
Interpersonal sensitivity	7 (17.5 %)	1.79 ± 0.72	1.65 ± 0.51
Depression	7 (17.5 %)	1.90 ± 0.77	1.5 ± 0.59
Anxiety	8 (20 %)	1.76 ± 0.74	1.39 ± 0.43
Anger and hostility	5 (12.5 %)	1.76 ± 0.75	1.48 ± 0.56
Phobic anxiety	7 (17.5 %)	1.52 ± 0.66	1.23 ± 0.41
Paranoid ideation	4 (10 %)	1.64 ± 0.62	1.43 ± 0.57
Psychoticism	5 (12.5 %)	1.57 ± 0.50	1.29 ± 0.42
Average	6.7 (16.7 %)		

The psychological status of patients was evaluated using the SCL-90 scale [17]. ^a^ *n* = the number of patients’ score outside the normal range of reference data (mean ± 1.96 SD)

## Discussion

In this study, we applied the full battery of standardized QST consisting of 13 parameters to patients with TMD pain and age-, gender-, and region-stratified healthy participants. The main finding was that 82.5 % of the patients presented somatosensory abnormalities in terms of loss or gain of somatosensory function in the painful facial regions, while the frequency of abnormality in the right hand region was 60.0 %, compared with 31.2 % in facial and 24.3 % in hand regions of the reference population. Mechanical tests were more often associated with abnormalities than thermal tests (Tables 1 and 3). The most frequent loss/gain score encountered was L0G2 (no somatosensory loss combined with gain of mechanical somatosensory function). Interestingly, this result corresponds with the score most frequently found in patients with trigeminal neuropathic pain and atypical odontalgia in a mainly Caucasian population [7, 18], but was much less common in other non-trigeminal neuropathic pain conditions [7]. Some TMD pain patients in the present study did not show somatosensory abnormalities during QST at the standard testing areas. This phenomenon could have been due to the standardization of test sites in our study. QST was performed over the most painful sites of the jaw muscles or the temporomandibular joints in all patients, while pain in TMD can originate from 12 specified test sites in the temporalis and masseter muscles or temporomandibular joint per side [16]. Only a few studies have demonstrated somatosensory abnormalities in TMD patients [14, 19]. The important strengths of the present study were the standardized evaluation of the phenotypes in terms of somatosensory abnormalities in Chinese TMD pain patients using loss and gain scores based on a comprehensive QST protocol and Z-score transformation [9], Comparing indirectly between the results of the present study in a Chinese population and earlier studies in a mainly Caucasian patient population, it seems that gain of mechanical function (mechanical hyperalgesia) is the most frequent somatosensory abnormality for both Chinese and Caucasian TMD patients [14, 15].

### Somatosensory abnormalities according to the loss/gain system

The loss/gain coding system includes the evaluation of absolute (score outside 95 % CI of reference) and relative (side-to-side difference outside 95 % CI of reference) abnormalities [7, 18]. It has been suggested that using absolute abnormalities alone may be too conservative for detecting somatosensory abnormalities, mainly due to the large inter-individual variation in somatosensory sensitivity [7]. Involving the side-to-side difference in the evaluation increased the diagnostic sensitivity by 2.5–25.0 % across different parameters in our study (Fig. 3), and did not change their pattern, which is in line with an earlier study [7].

Like the studies conducted by Maier et al. and Baad-Hansen et al. [7, 18], we also detected some somatosensory abnormalities in the healthy group, a total of 31.2 % showing one or more values outside the 95 % CI (Table 3). This frequency may seem high, but is actually lower than would be expected based on simple calculation of the probability of a healthy person having at least 1 of 11 values outside the 95 % CI (1–0.95^11^ = 43.1 %) [18]. The standardized QST and loss/gain coding system enables standardized evaluation of somatosensory changes in conditions and diseases with sensory disorders.

Pfau’s study demonstrated differences comparing a TMD patient group and control group for CPT, PPT, MPT, MPS and MDT with TMD patients being more sensitive to painful stimuli, but less sensitive to tactile stimulation on a group level [14]. In Kothari’s study, a total of 85.3 % of the TMD patients exhibited one or more somatosensory abnormalities at the most painful site. The most frequent somatosensory abnormalities in terms of gain of function were hyperalgesia to blunt pressure, hyperalgesia to pinprick-evoked pain, increased wind-up and heat and cold hyperalgesia [15]. The most frequent somatosensory abnormalities in terms of loss of function were observed for nonpainful thermal and mechanical submodalities in the TMD patients. Hypoalgesia to pinprick-evoked pain was detected in 2.9 % (MPS) of the TMD patients [15]. As the loss/gain system was not adopted by these previous studies, it is difficult to compare their results directly with the present study. Overall, it seems that the present Chinese/East Asian sample of TMD patients presented similar somatosensory abnormalities as the Caucasian/Western samples of TMD patients evaluated by same QST protocol, even though we have previously shown ethnic differences in somatosensory functions between healthy Chinese and healthy Caucasians [10].

### Somatosensory abnormalities in extra-trigeminal regions

Earlier studies investigating the pain sensitivity of TMD pain patients in extra-trigeminal regions reported increased experimentally-evoked pain in non-facial areas [9, 14, 20]. In this study, significant differences between groups in the extra-trigeminal control site (the dorsum of the right hand) were also detected, with TMD pain patients having more frequent somatosensory abnormalities than healthy controls (Additional file 1: Table S4), and the pattern of these abnormalities was the same as at the painful sites, but at a lower frequency. The lower frequency in the hand region may mainly be due to the lack of consideration of relative abnormalities (Table 3*vs* Additional file 1: Table S4). Somatosensory abnormalities in extra-trigeminal region may suggest that central mechanisms are involved in the pathophysiology of TMD pain [7, 21]. It has been suggested that generalized up-regulation of central responsiveness to aversive stimulation may constitute a pathophysiological mechanism contributing to myofascial pain in TMD patients [9, 20]. Disturbance of the endogenous opioid system in TMD pain patients with myalgia has been suggested, based on a deficit in pain inhibition by painful ischemic stimulation [22].

### Mechanism-based classification of TMD pain

The pain mechanisms underlying TMD are not fully understood, which complicates the diagnosis, treatment, and development of targeted analgesics. For individual patients, the impairment of joint or muscles, or pain duration are not consistent with the somatosensory changes [11], and the phenotype of somatosensory changes may not always be distinct across different subgroups of TMD patients [14]. Treatment strategies could be improved if quantitative biomarkers could be developed to phenotype patients with TMD pain in order to design individualized management. Standardized QST and statistical procedures (z-transform by individual patients and the loss/gain coding system) has the potential to be used for phenotyping TMD pain patients [4, 7, 13].

In a randomized, double-blind, placebo-controlled, and phenotype-stratified study, 83 peripheral neuropathic pain patients were tested by the same QST protocol used in the present study, and patients were grouped into an irritable nociceptor phenotype and non-irritable nociceptor phenotype according to the QST results [23]. The irritable nociceptor phenotype was defined as preserved small-fiber function (cold, warm, and pinprick sensitivity) together with hyperalgesia, and the non-irritable nociceptor phenotype was defined as deafferentation type, which was dominated by sensory loss. The results indicated that oxcarbazepine was more efficacious for relief of peripheral neuropathic pain in patients with the irritable vs the non-irritable nociceptor phenotype [23].

The concept of mechanism-based management of TMD is supported by our data: different patients suffering from the same clinical disorder presented different phenotypes of somatosensory abnormalities. The highest rate of abnormality was L0G2 (no somatosensory loss with gain of mechanical somatosensory function), which is consistent with Baad-Hansen’s report in atypical odontalgia patients [18]. In contrast to conventional group comparisons, the DFNS recommends the approach of allowing clinical judgments on a single-case basis [7, 18]. Given that some TMD patients show increased, while others show decreased responses, group mean comparisons could give false-negative normal values. Somatosensory profiling combining many QST parameters increases the likelihood of detecting an abnormality in any given patient, but also the risk of false-positive results. Given the increasing patient demand for cost-effective, evidence-based management of TMD pain, identifying the characteristics of individual patients is critical to patient-centered and individualized care.

### Limitations of this study

A limitation of this study was the differences in the test sites between the two groups: the skin overlying the infraorbital and mental nerve regions for healthy controls, but the painful sites for TMD patients. However, the DFNS has similarly used data from the hand region to represent the upper body and data from the foot region to represent the lower body, and we suggest the reference material in this study to be equally appropriate [4, 7]. Another limitation could be that the sample size of the healthy controls did not allow for extensive age-stratification related to somatosensory changes. Due to the small sample size in each group, myalgia and arthralgia patients were evaluated as one group for this exploratory analysis. These need to be improved in future multicenter studies.

## Conclusions

This is the first study using the full battery of QST tests and statistical protocol recommended by the German Research Network on Neuropathic Pain in the orofacial region, to assess the somatosensory function of Chinese patients with painful temporomandibular disorders and Chinese healthy controls. Furthermore, the TMD diagnoses were based on a validated and reproducible diagnostic system (DC/TMD). Somatosensory abnormalities were detected in 82.5 % for painful sites and 60.0 % for the extra-trigeminal site in TMD pain patients, most frequently in the form of somatosensory gain to nociceptive mechanical stimuli for both regions, suggesting a more generalized sensitization in the patients. TMD pain patients presented differences in somatosensory profiles, which may support the concept of individualized pain mechanisms-based management.

## Additional file

Additional file 1:
**Table S1.** Quantitative sensory testing absolute data from 70 healthy participants for each parameter stratified by age group, gender, and test site: Mean (SD). **Table S2.** Quantitative sensory testing relative data from 70 healthy participants for each parameter stratified by gender, age group, and test site: Mean (SD). **Table S3.** Frequency (%) of TMD patients and healthy reference participants presenting with hand site z-score values outside the reference 95% confidence interval ( -1.96 < z < 1.96). **Table S4.** Loss and gain distribution in the right hand in temporomandibular disorder (TMD) patients and the reference group. (DOCX 34 kb)

## Abbreviations

CDT: cold detection threshold
CPT: cold pain threshold
DC/TMD: diagnostic criteria for TMD
DFNS: German Research Network on Neuropathic Pain
DMA: dynamic mechanical allodynia
HPT: heat pain threshold
MDT: mechanical detection threshold
MPS: mechanical pain sensitivity
MPT: mechanical pain threshold
PHS: paradoxical heat sensation
PPT: pressure pain threshold
QST: quantitative sensory testing
TMD: temporomandibular disorders
TSL: thermal sensory limen
VDT: vibration detection threshold
WDT: warmth detection threshold
WUR: windup ratio
