# Supplementary material for: Somatosensory abnormalities in Chinese patients with painful temporomandibular disorders
Source: J Headache Pain. 2016 Apr 12;17:31. doi: 10.1186/s10194-016-0632-y (PMC4829566; doi:10.1186/s10194-016-0632-y)
Supplement: Additional file 1: — Table S1. Quantitative sensory testing absolute data from 70 healthy participants for each parameter stratified by age group, gender, and test site: Mean (SD). Table S2. Quantitative sensory testing relative data from 70 healthy participants for each parameter stratified by gender, age group, and test site: Mean (SD). Table S3. Frequency (%) of TMD patients and healthy reference participants presenting with hand site z-score values outside the reference 95% confidence interval ( -1.96 < z < 1.96). Table S4. Loss and gain distribution in the right hand in temporomandibular disorder (TMD) patients and the reference group. (DOCX 34 kb) [file 10194_2016_632_MOESM1_ESM.docx]

**Additional file 1**

**Table S1.** Quantitative sensory testing absolute data from 70 healthy participants for each parameter stratified by age group, gender, and test site: Mean (SD)

|  |  | CDT(*△*℃) | WDT(*△*℃) | TSL(℃) | HPT(℃) | CPT(℃) | MDT(mN) | MPT(mN) | MPS | WUR | VDT(scale) | PPT(KPa) |
| --- | --- | --- | --- | --- | --- | --- | --- | --- | --- | --- | --- | --- |
| female |  |  |  |  |  |  |  |  |  |  |  |  |
| 21-40 years (n=16) | Hand | -1.29(0.65) | 1.87(0.78) | 3.62(1.40) | 38.88(2.59) | 26.29(5.54) | 1.60(1.61) | 131.69(65.44) | 1.11(0.83) | 4.02(2.32) | 7.66(0.43) | 182.85(58.15) |
|  | Infraorbital | -0.83(0.65) | 1.04(0.37) | 2.23(0.99) | 36.51(1.71) | 26.80(3.55) | 0.12(0.06) | 70.56(38.37) | 1.58(0.95) | 4.15(2.26) | 7.30(0.47) | 133.08(30.62) |
|  | Mental | -0.79(0.28) | 1.10(0.47) | 2.23(0.85) | 37.71(2.64) | 26.79(5.29) | 0.11(0.09) | 66.38(43.28) | 1.75(1.55) | 3.35(2.00) | 7.43(0.47) | 113.52(38.52) |
| 41-70 years (n=20) | Hand | -1.58(0.50) | 2.28(0.77) | 4.28(1.56) | 39.98(3.60) | 22.42(7.65) | 2.71(2.94) | 124.77(93.89) | 1.29(0.91) | 2.21(1.29) | 7.77(0.32) | 228.24(76.14) |
|  | Infraorbital | -0.94(0.42) | 1.21(0.33) | 2.76(1.22) | 38.48(3.23) | 22.21(6.28) | 0.13(0.08) | 76.37(62.93) | 2.35(1.21) | 2.56(1.85) | 7.48(0.42) | 176.65(54.16) |
|  | Mental | -0.97(0.32) | 1.49(0.50) | 2.80(1.34) | 39.33(3.09) | 22.01(7.63) | 0.10(0.03) | 68.04(69.95) | 2.90(1.89) | 2.50(1.95) | 7.62(0.41) | 142.65(43.99) |
| male |  |  |  |  |  |  |  |  |  |  |  |  |
| 21-40 years (n=16) | Hand | -1.46(0.55) | 2.28(0.82) | 4.51(1.72) | 40.54(3.58) | 24.85(5.99) | 2.39(3.05) | 196.37(139.02) | 1.01(1.18) | 2.60(1.97) | 7.64(0.44) | 276.48(159.59) |
|  | Infraorbital | -0.71(0.26) | 1.02(0.32) | 2.44(0.83) | 36.71(2.10) | 25.63(5.78) | 0.11(0.03) | 111.59(64.19) | 1.83(1.78) | 2.68(1.77) | 7.26(0.51) | 160.79(41.72) |
|  | Mental | -0.80(0.29) | 1.10(0.39) | 2.03(0.81) | 38.11(2.83) | 25.03(5.88) | 0.11(0.04) | 92.43(74.77) | 1.78(1.06) | 3.15(2.49) | 7.38(0.69) | 163.59(55.13) |
| 41-70 years (n=18) | Hand | -2.50(1.57) | 3.50(1.72) | 7.27(3.31) | 42.57(3.33) | 20.77(6.95) | 5.31(4.18) | 173.81(119.85) | 1.60(1.94) | 2.92(1.52) | 7.54(0.53) | 269.91(70.81) |
|  | Infraorbital | -1.08(0.37) | 1.33(0.28) | 3.69(1.79) | 38.40(2.92) | 21.35(5.62) | 0.17(0.12) | 100.08(85.72) | 2.42(1.89) | 2.74(1.30) | 7.38(0.53) | 207.26(66.27) |
|  | Mental | -1.13(0.49) | 1.60(0.68) | 3.53(1.48) | 40.53(3.44) | 20.91(6.51) | 0.16(0.13) | 88.79(94.62) | 2.30(1.94) | 3.21(1.97) | 7.52(0.59) | 158.04(43.29) |

CDT: cold detection threshold; WDT: warmth detection threshold; TSL: thermal sensory limen; PHS: paradoxical heat sensation; CPT: cold pain threshold; HPT: heat pain threshold; MDT: mechanical detection threshold; MPT: mechanical pain threshold; MPS: mechanical pain sensitivity; DMA: dynamic mechanical allodynia; WUR: windup ratio; VDT: vibration detection threshold; PPT: pressure pain threshold. △ = difference from the baseline temperature 32 °C.

**Table S2.** Quantitative sensory testing relative data from 70 healthy participants for each parameter stratified by gender, age group, and test site: Mean (SD)

|  |  | CDT(℃) | WDT(℃) | TSL(℃) | HPT(℃) | CPT(℃) | MDT(mN) | MPT(mN) | MPS | WUR | VDT(scale) | PPT(KPa) |
| --- | --- | --- | --- | --- | --- | --- | --- | --- | --- | --- | --- | --- |
| female |  |  |  |  |  |  |  |  |  |  |  |  |
| 21-40 years (n=16) | Hand | 0.25(0.31) | 0.53(0.54) | 0.72(0.56) | 1.26(1.07) | 1.42(1.22) | 1.14(1.47) | 37.03(32.19) | 0.26(0.36) | 1.41(1.44) | 0.16(0.20) | 44.61(32.05) |
|  | Infraorbital | 0.32(0.19) | 0.31(0.20) | 0.54(0.34) | 1.14(1.08) | 1.37(1.63) | 0.02(0.02) | 18.41(16.92) | 0.42(0.41) | 1.37(1.32) | 0.19(0.18) | 7.90(9.00) |
|  | Mental | 0.08(0.08) | 0.26(0.19) | 0.29(0.23) | 0.77(0.61) | 0.56(0.44) | 0.05(0.11) | 24.58(26.46) | 0.41(0.27) | 1.09(1.52) | 0.20(0.18) | 15.43(15.28) |
| 41-70 years (n=20) | Hand | 0.29(0.25) | 0.66(0.64) | 0.95(0.79) | 1.11(0.95) | 2.55(2.85) | 2.79(3.35) | 63.74(68.68) | 0.65(0.60) | 0.58(0.63) | 0.15(0.19) | 40.93(35.07) |
|  | Infraorbital | 0.22(0.19) | 0.24(0.25) | 0.64(0.46) | 2.22(2.20) | 2.12(1.56) | 0.04(0.06) | 26.39(27.20) | 0.53(0.52) | 0.61(0.97) | 0.20(0.20) | 32.61(23.21) |
|  | Mental | 0.30(0.23) | 0.31(0.25) | 0.42(0.37) | 1.38(1.20) | 1.46(1.21) | 0.02(0.02) | 27.68(46.14) | 1.03(1.07) | 1.08(1.48) | 0.19(0.21) | 26.21(23.07) |
| male |  |  |  |  |  |  |  |  |  |  |  |  |
| 21-40 years (n=16) | Hand | 0.41(0.36) | 0.94(0.69) | 0.82(0.80) | 1.17(0.89) | 1.65(1.13) | 2.08(2.14) | 42.09(35.49) | 0.57(1.22) | 0.93(0.91) | 0.16(0.16) | 57.77(37.06) |
|  | Infraorbital | 0.12(0.14) | 0.23(0.28) | 0.64(0.42) | 0.92(0.96) | 1.65(1.80) | 0.02(0.02) | 47.14(38.14) | 0.69(1.49) | 1.21(1.63) | 0.31(0.31) | 18.46(25.99) |
|  | Mental | 0.15(0.14) | 0.20(0.23) | 0.32(0.19) | 1.15(0.96) | 0.81(0.93) | 0.02(0.02) | 17.28(20.09) | 0.39(0.40) | 1.03(1.31) | 0.12(0.16) | 30.14(23.32) |
| 41-70 years (n=18) | Hand | 0.80(0.82) | 1.32(1.03) | 2.69(2.26) | 0.98(0.87) | 2.65(2.33) | 3.17(3.57) | 59.69(47.09) | 0.48(0.58) | 0.83(0.93) | 0.23(0.19) | 54.45(57.43) |
|  | Infraorbital | 0.25(0.22) | 0.14(0.12) | 0.75(0.68) | 1.27(1.55) | 2.17(1.89) | 0.10(0.11) | 41.56(52.83) | 0.77(0.90) | 0.90(1.09) | 0.24(0.18) | 36.16(22.91) |
|  | Mental | 0.25(0.29) | 0.48(0.44) | 0.65(0.52) | 1.28(1.28) | 2.34(2.16) | 0.08(0.12) | 39.05(83.17) | 0.98(1.35) | 0.88(0.76) | 0.14(0.16) | 30.37(21.83) |

Relative data indicates absolute values of side-to-side differences at the same test region. CDT: cold detection threshold; WDT: warmth detection threshold; TSL: thermal sensory limen; PHS: paradoxical heat sensation; CPT: cold pain threshold; HPT: heat pain threshold; MDT: mechanical detection threshold; MPT: mechanical pain threshold; MPS: mechanical pain sensitivity; DMA: dynamic mechanical allodynia; WUR: windup ratio; VDT: vibration detection threshold; PPT: pressure pain threshold.

**Table S3.** Frequency (%) of TMD patients and healthy reference participants presenting with hand site z-score values outside the reference 95% confidence interval ( -1.96 < z < 1.96).

|  | Reference group (n=70) | |  | Patient group (n=40) | |
| --- | --- | --- | --- | --- | --- |
|  | < -1.96 n(%) | >1.96  n(%) |  | < -1.96  n(%) | >1.96  n(%) |
| CDT | 4(5.7%) | 0(0.0%) |  | 4(10.0%) | 1(2.5%) |
| WDT | 5(7.1%) | 0(0.0%) |  | 4(10.0%) | 0(0.0%) |
| TSL | 2(2.8%) | 0(0.0%) |  | 3(7.5%) | 0(0.0%) |
| CPT | 3(4.3%) | 0(0.0%) |  | 3(7.5%) | 0(0.0%) |
| HPT | 1(1.4%) | 0(0.0%) |  | 0(0.0%) | 1(2.5%) |
| MDT | 2(2.8%) | 0(0.0%) |  | 0(0.0%) | 0(0.0%) |
| MPT | 4(5.7%) | 0(0.0%) |  | 5(12.5%) | 0(0.0%) |
| MPS | 0(0.0%) | 5(7.1%) |  | 0(0.0%) | 10(25.0%) |
| WUR | 0(0.0%) | 1(1.4%) |  | 0(0.0%) | 8(20.0%) |
| VDT | 2(2.8%) | 0(0.0%) |  | 2(5.0%) | 0(0.0%) |
| PPT | 4(5.7%) | 0(0.0%) |  | 4(10.0%) | 1(2.5%) |
| Mean all | 3.51(2.4%) | 0.78(1.1%) |  | 2.27(5.7%) | 1.91(4.8%) |

CDT: cold detection threshold; WDT: warmth detection threshold; TSL: thermal sensory limen; PHS: paradoxical heat sensation; CPT: cold pain threshold; HPT: heat pain threshold; MDT: mechanical detection threshold; MPT: mechanical pain threshold; MPS: mechanical pain sensitivity; DMA: dynamic mechanical allodynia; WUR: windup ratio; VDT: vibration detection threshold; PPT: pressure pain threshold.

**Table S4.** Loss and gain distribution in the right hand in temporomandibular disorder (TMD) patients and the reference group

| Loss | Gain | | | | All |
| --- | --- | --- | --- | --- | --- |
|  | G0 (None) | G1 (Thermal) | G2 (Mechanical) | G3 (Both) |  |
| TMD patients (n=40) |  |  |  |  |  |
| L0 (None) | 16(40.0%) | 1(2.5%) | 11(27.5%) | 0(0.0%) | 28(70.0%) |
| L1 (Thermal) | 5(12.5%) | 0(0.0%) | 5(12.5%) | 0(0.0%) | 10(25.0%) |
| L2 (Mechanical) | 1(2.5%) | 0(0.0%) | 1(2.5%) | 0(0.0%) | 2(5.0%) |
| L3 (Both) | 0(0.0%) | 0(0.0%) | 0(0.0%) | 0(0.0%) | 0(0.0%) |
| All | 22(55.0%) | 1(2.5%) | 17(42.5%) | 0(0.0%) | 40(100%) |
| Reference (n=70) |  |  |  |  |  |
| L0 (None) | 53(75.7%) | 0(0.0%) | 3(4.3%) | 0(0.0%) | 56(80.0%) |
| L1 (Thermal) | 9(12.9%) | 0(0.0%) | 1(1.4%) | 0(0.0%) | 10(14.3%) |
| L2 (Mechanical) | 3(4.3%) | 0(0.0%) | 1(1.4%) | 0(0.0%) | 4(5.7%) |
| L3 (Both) | 0(0.0%) | 0(0.0%) | 0(0.0%) | 0(0.0%) | 0(0.0%) |
| All | 65(92.8%) | 0(0.0%) | 5(7.1%) | 0(0.0%) | 70(100%) |

Sensory abnormality coding system [7]: hypoesthesia to thermal stimuli (loss of detection in the cold or warm detection threshold) was coded as L1, and hypoesthesia to mechanical stimuli (loss of detection in mechanical or vibration detection threshold) as L2. Signs of hyperalgesia to thermal stimuli (gain-of-function in heat or cold pain threshold) were coded as G1, and hyperalgesia to mechanical stimuli (gain-of-function in mechanical pain threshold or sensitivity, dynamic mechanical allodynia, or pressure pain threshold) as G2. When both thermal and mechanical abnormalities were present, L3 or G3 were defined. Normal values were coded as zero.
